# Fungal Pathogenesis at the Host–Pathogen Interface: Immune Recognition, Evasion, Antifungal Resistance, and Emerging Therapies

**DOI:** 10.3390/tropicalmed11100279

**Published:** 2026-09-30

**Authors:** Esteban Ortiz-Prado, Jorge Vasconez-Gonzalez, Karla Alejandra Cuenca, Fiorella Núñez-Loor, María Camila Acosta-Muñoz, Dario Alejandro Mesias, Karen Villarreal-Burbano, Marlon Arias-Intriago, Juan S. Izquierdo-Condoy

**Affiliations:** One Health Research Group, Faculty of Health Science, Universidad de Las Americas, Quito 170137, Ecuador

**Keywords:** fungal pathogenesis, host–fungus interactions, immune recognition, immune evasion, antifungal resistance, immunotherapy, fungal vaccines

## Abstract

Severe fungal diseases are an increasingly important yet underrecognized cause of global morbidity and mortality, with the greatest impact among immunocompromised individuals and populations with limited access to timely diagnosis and treatment. Disease outcome is determined by a dynamic host–fungus interface in which innate pattern-recognition receptors sense fungal cell-wall ligands and coordinate complement activation, phagocyte responses, and cytokine signaling, while adaptive Th1- and Th17-mediated immunity supports fungal containment and clearance. Pathogenic fungi counter these defenses through cell-wall remodeling and antigen masking, morphological transitions, capsule and biofilm formation, resistance to oxidative stress, complement subversion, interference with phagosome maturation, intracellular persistence, and colonization of immune-privileged sites. These mechanisms are deployed in species-specific combinations by *Candida*, *Aspergillus*, *Cryptococcus*, *Histoplasma*, *Coccidioides*, *Blastomyces*, *Mucorales*, and other medically important fungi, shaping tissue tropism, persistence, dissemination, and disease severity. Therapeutic control is further undermined by intrinsic and acquired resistance to azoles, echinocandins, and polyenes through target modification, efflux-pump upregulation, compensatory cell-wall remodeling, and altered ergosterol biosynthesis. This review integrates the mechanisms of antifungal immune recognition with common and species-specific evasion strategies and discusses antifungal resistance, emerging host-directed therapies, and vaccine platforms. Defining how fungal adaptation intersects with immune dysfunction and drug resistance may reveal conserved therapeutic targets and support rational combination strategies. Translating these insights into effective antifungal therapies, safe immunomodulatory interventions, and broadly protective vaccines will be essential to improve clinical outcomes and strengthen preparedness, particularly in resource-limited settings.

## 1. Introduction

In medicine, there is a particularly complex and challenging issue to address: fungal infections. These types of infections are a major global health problem, with an estimated 13 million cases and around 1.5 million deaths occurring annually [1]. These infections encompass a broad spectrum of clinical presentations, ranging from superficial cutaneous mycoses to severe, often treatment-refractory systemic infections, which can be fatal depending on host immune status and pathogen virulence [2,3]. Severe fungal infections are broadly categorized into two groups: opportunistic mycoses, including candidiasis, cryptococcosis, and invasive infections such as aspergillosis and zygomycosis, and endemic mycoses, which are often geographically restricted, but can lead to life-threatening disease [4,5]. In the case of *Coccidioides*, the main route of transmission is through the respiratory tract, that is, via inhalation. However, rare and infrequent cases of transmission have been reported through organ transplantation and also through skin inoculation [6]. *Histoplasma* is transmitted through the inhalation of microconidia or hyphal fragments. There is no person-to-person transmission; however, in very rare cases, it can occur through organ transplantation [7]. In the case of *Blastomyces*, it is capable of causing infections when its conidia become airborne and are inhaled [8].

The primary modes of transmission include direct contact particularly in the case of dermatophytes, which secrete proteolytic enzymes to invade keratinized tissues, as well as the inhalation of spores or conidia, which can lead to pulmonary infections [5]. On the other hand, an endogenous fungal infection can be acquired through colonization or reactivation of a fungus from a latent infection; fungi can also gain access to host tissues through traumatic implantation [9,10]. It is necessary to emphasize that fungal zoonoses have the potential to be transmitted through bites, scratches, contact with suppurative skin lesions, and even through the skin or hair of infected animals [11].

Once inside the host, pathogenic fungi employ a range of sophisticated strategies to evade immune surveillance and establish infection. As eukaryotic organisms, fungi share many cellular features with their hosts, which not only complicates immune recognition but also facilitates their capacity to invade diverse tissues and undergo morphogenetic transitions that enhance survival and persistence within the host environment [12,13]. These immune evasion strategies encompass a wide array of molecular and cellular mechanisms that pathogenic fungi have evolved to circumvent host defenses. Among the most studied are the formation of robust biofilms that inhibit antifungal penetration and immune cell access; strategic modifications of cell surface components such as β-glucans and mannoproteins, which effectively mask pathogen-associated molecular patterns (PAMPs); and the modulation or suppression of immune signaling pathways [14,15,16]. Furthermore, fungi can survive intracellularly within macrophages and dendritic cells by interfering with phagosome maturation and resisting oxidative stress. Certain species, like *Cryptococcus neoformans*, can induce the formation of titan cells or manipulate host pattern recognition receptors (PRRs) to trigger anti-inflammatory responses, ultimately weakening host immunity [17]. These strategies affect cell wall modifications, capsule formation, dimorphic switching, and biofilm production (Figure 1), all of which impair immune recognition and clearance [14,18]. Some fungi can even interfere with phagocytosis, evade immune cells or actively suppress antimicrobial production [15,19]. Collectively, these tactics allow fungi not only to avoid detection but also to persist and proliferate within immunocompromised or otherwise susceptible hosts. 

It has been proposed that these mechanisms developed in response to selective pressure from the host, which drove the evolution of various adaptations, including adhesion to host surfaces, competitive interaction with the microbiome, ability to obtain nutrients, adaptation to the physiological temperatures of the organism, and mechanisms to evade the immune system response [20]. Given these complexities, a deeper understanding of fungal immune evasion mechanisms is essential for advancing diagnostic approaches, designing targeted antifungal therapies, and improving host-directed treatments [21,22]. This review analyzes the immune evasion strategies of pathogenic fungi and their impact on fungal pathogenesis, as well as the challenges for treatment and potential therapeutic strategies.

## 2. Host Immune Response to Fungi

### 2.1. Innate Immune Response to Fungi

The innate immune system represents the first line of defense against fungal infections, which involves cells such as macrophages, dendritic cells, and neutrophils. The cell wall of these organisms contains PAMPs, which are recognized by pattern recognition receptors (PRRs) expressed on cells of the innate immune system and epithelial cells. These receptors include C-type lectin receptors (CLRs), RIG-I-like receptors, Toll-like receptors, and NOD-like receptors [23,24,25]. On the other hand, epithelial cells have the ability to initiate an immune response similar to that of cells of the myeloid lineage, thus playing an important role in the active recognition of microorganisms [26].

Toll-like receptors (TLRs) possess leucine-rich repeats in their extracellular domains, which are known to enhance their ability to bind a variety of fungal PAMPs. Additionally, the intracellular Toll/IL-1 receptor (TIR) domain has been described as responsible for initiating immune activation by triggering various signaling pathways. Among these is the activation of TLR2 by fungal components such as zymosan, which recruits adaptor proteins like MyD88, leading to the activation of NF-κB and the production of pro-inflammatory cytokines, essential for mounting an effective immune response [25]. CLRs including Dectin-1, Dectin-2, Mincle, DC-SIGN, and the mannose receptor (MR) recognize carbohydrate ligands like β-1,3-glucan, β-1,6-glucan, mannans, chitin, and melanin [23,25] (Figure 2).

Neutrophils are essential in defense against *Candida* spp. and *Aspergillus* spp., through phagocytosis and reactive oxygen species (ROS) production via the nicotinamide adenine dinucleotide phosphate (NADPH) oxidase complex. Macrophages contribute to controlling *Cryptococcus* spp. and *Histoplasma* spp. infections [27]. Dendritic cells act as a link between the innate and adaptive immune responses by processing and presenting fungal-derived antigens to CD4^+^ and CD8^+^ T lymphocytes. They also recognize fungal virulence factors based on morphology, allowing for an adaptive immune response tailored to the level of fungal pathogenicity [28]. Given the critical role of CLRs in fungal immunity, maintaining their proper regulation is essential for an effective immune response [28,29].

### 2.2. Adaptive Immune Response to Fungi

T helper (Th) cells orchestrate the adaptive antifungal immune response. Th1 cells are responsible for the production of IFN-γ, which is essential for the elimination of intracellular pathogens, while Th2 cells produce IL-5 and IL-13, which help eliminate certain fungi [30]. Th17 cells are critical for immunity against mucocutaneous fungal infections. IL-17 enhances neutrophil recruitment and activity and contributes to IgA production, which is essential for mucosal defense. Deficiency in IL-17 signaling has been associated with chronic mucocutaneous candidiasis [25]. 

CD4^+^ T lymphocytes play a fundamental role in coordinating the immune response, driving macrophage activation, stimulating B cell activity, and amplifying cytokine production to eradicate fungal pathogens [31]. In contrast, while CD8 T cells typically play a secondary role compared to CD4 T cells in antifungal defense, their significance emerges in certain contexts. It has been described that in immunocompromised patients with invasive pulmonary aspergillosis, a reduction in the number of CD8 T cells is associated with poorer clinical outcomes [25] (Figure 3).

The immune response to fungal infections is characterized by a complex interaction between the host and the pathogen, requiring coordination between innate and adaptive immunity for effective infection control. The ability of fungi to evade recognition by the immune system and modulate host responses presents significant challenges, particularly in immunocompromised populations. 

## 3. Immune Evasion Strategies

### 3.1. Fungal Cell Wall Modification 

It is well known that these microorganisms have the ability to thrive in challenging environments, as well as to undergo rapid changes thanks to the activation of different pathways that allow them to adapt to stress. This is largely due to the cell wall, which can modify its composition and structure, providing these organisms with great adaptive capacity and the ability to survive under adverse conditions [16,32].

The inner layer of the cell wall of many fungi, especially pathogenic ones, contains chitin and β-glucan, which form the main structure of the fungal cell wall. On the other hand, the outer layer of the cell wall varies among different fungal species and can mask the internal polysaccharides, thereby hindering their recognition by the immune system [16,33]. The outer layers of the cell wall in *Candida*, *Aspergillus*, *Histoplasma*, and *Cryptococcus* are generally able to prevent pattern recognition receptors from binding to ligands buried within the inner layers [16]. In the case of *Candida albicans*, the outer layer of the cell wall contains mannoproteins modified with glycosylphosphatidylinositol (GPI) and cross-linked with β-1,6-glucans [33]. *Cryptococcus neoformans*, in turn, has α-1,3-glucan, β-1,3- and β-1,6-glucans, chitin, chitosan, mannoproteins, and other GPI-anchored proteins in its cell wall; additionally, it is notable for the presence of a polysaccharide capsule that surrounds the outer part of its cell wall [33,34]. In contrast, *Aspergillus fumigatus* lacks β-1,3-glucan and chitin in the outer layer of its cell wall [33].

The immune system is able to easily recognize β-glucan, triggering an effective response to fight the infection. For this reason, masking β-glucan is one of the main mechanisms used by fungi to avoid immune recognition. Additionally, this mechanism may provide resistance to complement system activation through the classical and alternative pathways, ultimately reducing the effectiveness of the immune response [33]. *Histoplasma capsulatum* masks its β-glucan with α-glucan, while *Paracoccidioides brasiliensis* actually converts its (1,3)-β-glucans into (1,3)-α-glucans in the host. In general, the outer mannan layer of many fungal cells shields the highly immunogenic β-1,3-glucan from the Dectin-1 receptor. In the case of *Histoplasma* cells, endo-β-1,3-glucanase is released to eliminate β-1,3-glucans from the cell surface, thereby preventing their recognition [16,18].

Regarding chitin, it can interfere with the recognition of *Candida* species by peripheral blood mononuclear cells and murine macrophages, thereby reducing cytokine production. It has also been reported that chitin alters nitric oxide production by inducing arginase-1 in macrophages, which in turn results in a decrease in the antimicrobial activity of these cells [35].

Melanin is a high-molecular-weight pigment that provides mechanical strength to the fungal cell wall, resistance to enzymatic degradation, and UV protection. It is known to influence the host immune response and inhibit phagocytosis, as melanized fungi have been observed to be more resistant to phagocytosis compared to non-melanized cells [18,36]. In *Cryptococcus neoformans*, this resistance mechanism has been associated with changes in the surface electrical charge of fungal cells, which may hinder their recognition and subsequent phagocytosis by macrophages. Furthermore, a considerable reduction in nitric oxide production by these immune cells has been observed. In *Aspergillus*, melanin inhibits apoptosis in macrophages that have phagocytosed melanized conidia [37,38]. Melanin is an important virulence factor, playing a critical role in the dissemination of *Cryptococcus* to the host brain, and in the case of *C. neoformans*, it has been linked to the spread of yeast cells from the lungs to other organs since it influences the host immune response and inhibits phagocytosis [33].

Regarding the capsule, described in a limited number of fungi, it provides protection from the environment and serves as a source of virulence factors. Additionally, it has antiphagocytic properties, plays a role in the suppression of T lymphocyte proliferation, and induces the anti-inflammatory cytokine IL-10. It also plays a role in the reduction in pro-inflammatory cytokines and survival within the macrophage environment. Furthermore, it prevents the formation of neutrophil extracellular traps (NETs) through the capsular component GXM [18].

### 3.2. Immune Evasion Mechanisms Through Morphological and Structural Adaptations

A remarkable strategy that some pathogenic fungi have acquired to avoid phagocytosis is evading engulfment by phagocytic cells through volume expansion, either by increasing cell size or undergoing morphogenesis. Examples include *C. neoformans* Titan cells, or a morphotype switch, as seen in the dimorphic fungi *Coccidioides immitis* and *C. posadasii*, which exist as hyphae in the environment but form spherules up to 120 µm in diameter inside the host too large to be phagocytosed by macrophages [18].

Another mechanism is the evasion of neutrophil extracellular traps (NETs). Fungi have developed numerous strategies that allow them to resist capture or elimination by these structures. *Cryptococcus neoformans* possesses polysaccharides that provide protection against neutrophils and other host immune cells. *Candida albicans* has genes associated with its polysaccharide matrix that regulate resistance to NETs, while *Candida glabrata* delays the release of NETs [39].

Inhibition of complement activity is another antiphagocytic mechanism employed by various fungi. For example, *Candida albicans* produces secreted aspartyl proteases (Saps), including Sap1, Sap2, and Sap3, which can degrade and inactivate the complement components C3b, C4b, and C5, thereby reducing complement-mediated damage and helping the fungus evade this host immune defense mechanism [40]. *Aspergillus* species can bind complement inhibitors such as FH, FHL-1, and C4BP, blocking the progression of the complement cascade. They are also capable of using pigments located on the surface of conidia to conceal C3 binding sites, thereby avoiding opsonization and complement attack [41]. It has been observed that several types of fungi possess proteins that bind to plasminogen, leading to the production of plasmin. Once activated, plasmin degrades complement effectors and inhibits the activity of C3 and C5 convertases, which results in the inactivation of C3b and ultimately impairs the activation of the complement system [41].

An interesting strategy employed by *Candida albicans* is the filamentation of phagocytosed cells, which leads to the piercing of immune cells, allowing the pathogen to escape. It can also form hyphae outside of macrophages to avoid phagocytosis and limit the recruitment of additional macrophages. Meanwhile, *Candida lusitaniae* evades macrophage activity by producing cellular chains, thereby avoiding recognition by the immune system [41,42]. 

Several pathogenic fungi, such as *Candida albicans*, *Cryptococcus neoformans*, *Blastomyces dermatitidis*, and *Coccidioides posadasii*, can inhibit nitric oxide (NO) production by macrophages, which may be mediated through the downregulation of macrophage iNOS mRNA expression [15]. Additionally, these pathogens may utilize enzymatic mechanisms—such as superoxide dismutases (SODs), catalases (CATs), and peroxiredoxins (PRXs)—as well as non-enzymatic defenses like melanin, mannitol, and trehalose to maintain redox homeostasis within the host cell, resist oxidative stress, or repair damage [41].

Breda et al. investigated how *Fonsecaea pedrosoi* modulates the host immune response through interactions with neutrophils. By administering an anti-Gr1 antibody to induce temporary neutropenia in mice, they observed that animals with reduced neutrophil counts exhibited a lower fungal burden in the liver and spleen during both early and late stages of infection [43]. These mice also showed decreased production of *F. pedrosoi*-specific antibodies and increased T cell proliferation. These findings suggest that under normal conditions, *F. pedrosoi* induces a polymorphonuclear myeloid-derived suppressor cell (PMN-MDSC) phenotype in neutrophils, which suppresses T cell expansion and promotes chronic infection. The study concludes that the host’s immune response to the fungus is enhanced when these “regulatory” neutrophils are eliminated, highlighting their role in immune suppression [43].

Rieber et al., using both murine models and human patient samples, showed that infections caused by fungi such as *Aspergillus fumigatus* and *Candida albicans* promote the development of a specific subset of neutrophilic myeloid-derived suppressor cells (MDSCs). This process is mediated by the pattern recognition receptor Dectin-1 and its adaptor protein CARD9, as well as downstream Dectin-1 signaling pathways—particularly the generation of reactive oxygen species (ROS), caspase-8 activity, and interleukin-1 production. These MDSCs functionally suppress T cell and NK cell responses [44].

By employing these multifaceted strategies, fungal pathogens effectively evade immune recognition, suppress host defenses, and ensure their survival and dissemination within the host (Figure 4).

### 3.3. Biofilm Formation as a Multilayered Immune Evasion Strategy

Biofilms are structures formed by microorganisms that are encapsulated in a self-produced matrix of extracellular polymeric substances. Due to the high density of cells protected by this polymeric shield, they are impermeable to antimicrobials and immune cells, and this shield also hides them from host recognition [18,45]. In addition, they act as reservoirs of drug-resistant persister cells and dispersal cells, armed with virulence factors capable of evading the immune system [45].

The interaction between fungal biofilms and the immune system is a complex process. Evidence indicates that the presence of adherent peripheral blood mononuclear cells (PBMCs) promotes the ability of *C. albicans* to develop biofilms, potentially through the action of a soluble factor released by these immune cells. Additionally, the proinflammatory cytokine IL-17A was found to promote biofilm formation. Neutrophils have also been observed to fail in eradicating *Candida* biofilms and even suppress the activation of reactive oxygen species (ROS) [18,45,46]. These biofilm-favoring effects were mediated by β-glucans present in the extracellular matrix of the biofilm [18].

Another example of immune evasion mediated by biofilms is observed in *Cryptococcus* species, which produce an extracellular matrix rich in glucuronoxylomannan (GXM). This component is responsible for a multifaceted inhibition of neutrophil function, impairing chemotaxis, phagocytosis, NET formation, and antifungal activity [46]. Another important aspect of this mechanism employed by fungi lies in the formation of biofilms on implanted devices such as central venous catheters, urinary catheters, joint prostheses, and mechanical heart valves, which represent a significant cause of recurrent infections [47]. The formation of biofilms on the surface of implanted devices increases the patient’s susceptibility to infections due to the creation of an immunologically compromised zone, compounded by the fact that biofilms also exhibit limited pharmacological susceptibility [47,48].

### 3.4. Immune Evasion Through Colonization of Immune-Privileged Sites

The CNS is protected by the blood–brain barrier, which restricts the entry of immune cells and soluble factors, thereby limiting the immune response within the brain and spinal cord. This environment, combined with fewer immune cells compared to other tissues, makes the CNS an ideal target for certain fungal pathogens. *Cryptococcus neoformans*, for example, is capable of penetrating the blood–brain barrier, which typically prevents infections from reaching the brain. The fungus achieves this by weakening the vascular and cerebrovascular barriers, breaking down extravascular fibrin nets that are meant to contain and neutralize pathogens during local infections, and triggering protease systems that degrade host tissues. One critical mechanism involves the exploitation of the plasminogen activation system, wherein cryptococcal surface proteins bind plasminogen and convert it to plasmin, enabling proteolysis of host barriers and facilitating parenchymal invasion [49].

Similarly, the eye is another privileged site where immune responses are less efficient. The eye’s microenvironment employs mechanisms akin to immunological tolerance, such as anergy and regulatory T and B cells (Tregs and Bregs), which modulate both innate and adaptive immune responses [50]. This unique environment does not favor the robust immune responses typically seen in other tissues, such as the skin, gut, or lungs. In healthy individuals, strong immune responses in these tissues effectively eliminate infections and restore homeostasis. In contrast, the eye’s attenuated immune response often fails to eradicate infections or reduce intraocular inflammation effectively [50].

Immunological tolerance, a central concept in the eye’s immune environment, ensures that the immune system avoids attacking the body’s own cells. According to the self-discrimination theory, tolerance is maintained through mechanisms such as the deletion, anergy, or regulation of autoreactive immune cells (T cells and B cells). These mechanisms ensure that the immune system targets only foreign antigens while sparing the body’s own tissues. However, this tolerance also creates a vulnerability that pathogens can exploit, as seen in fungal infections of the eye [50].

## 4. Species-Specific Immune Evasion Mechanisms in Medically Relevant Fungi

### 4.1. Aspergillus spp.

The genus *Aspergillus* comprises numerous species, some of which are highly pathogenic and pose significant risks to human health. These fungi have developed a repertoire of immune evasion mechanisms to resist effective complement attack. One such mechanism is immune concealment through the formation of fungal abscesses, which act as physical shields that inhibit the diffusion of complement factors to the hypha [51]. In addition, surface melanin prevents recognition by complement components and reduces C3 deposition on conidia. Melanin also contributes to fungal defense by neutralizing reactive oxygen species (ROS) and reducing phagolysosomal acidification in alveolar macrophages, monocyte-derived macrophages, and neutrophils [51].

Another mechanism involves the acquisition of host-derived complement inhibitors, such as factor H (FH), factor H-like protein 1 (FHL-1), factor H-related protein 1 (FHR-1), and C4b-binding protein (C4BP), which bind to the fungal surface and negatively regulate complement activation [51]. In addition, the synthesis of proteolytic enzymes degrades complement proteins, thereby preventing the formation of antimicrobial effector molecules. Complement degradation leads to a marked reduction in fungal hyphae opsonization and impairs the phagocytosis of conidia by neutrophils and microglial cells [51].

### 4.2. Candida spp.

This microorganism has the ability to regulate the complement cascade by recruiting factor H, factor H-like protein 1 (FHL-1), C4b-binding protein (C4BP), and plasminogen to its surface. These complement regulators retain their functionality, allowing the fungus to modulate and even evade complement-mediated attack [52]. Plasminogen plays a role in suppressing complement activity and promoting the breakdown of extracellular matrix components, thereby facilitating tissue invasion by *Candida albicans* [52]. Another mechanism employed involves the degradation of complement proteins by fungal proteases. For instance, secreted aspartyl proteases cleave C3b, C4b, and C5, inhibit membrane attack complex (MAC) assembly, degrade factor H and its associated complement receptors CR3 and CR4 on macrophages, and cleave immunoglobulins including IgG and IgA, both of which are essential for complement activation [52]. pH-regulated antigen 1 (Pra1) binds to factor H, FHL-1, and plasminogen, thereby mediating complement evasion as well as extracellular matrix degradation [53]. Additionally, it has been reported that Pra1 binds to C4BP, thereby maintaining the complement-inhibitory activity and promoting immune evasion [54]. Phosphoglycerate mutase 1 also interacts with host factors such as factor H, FHL-1, and plasminogen, thereby contributing to immune evasion and the degradation of the extracellular matrix [55]. Furthermore, Saps can cleave human mannose-binding lectin (MBL) and collectin-11, two key recognition molecules involved in initiating the lectin pathway of the complement system. By degrading these molecules, Saps can interfere with and prevent activation of the lectin pathway [56]. *Candida albicans* is also capable of escaping from macrophages by permeabilizing macrophage membranes through candidalysin and by activating cell death pathways such as Gasdermin D-mediated pyroptosis [57]. Moreover, fungal cells have been shown to inhibit Natural Killer (NK) cells by manipulating the immune checkpoint receptor TIGIT through the adhesin proteins Als6, Als7, and Als9-2, which are capable of binding directly to TIGIT in an independent manner [58].

Och1 is a Golgi-localized α1,6-mannosyltransferase that plays a crucial role in N-glycan biosynthesis, which is involved in interactions between the host and fungal pathogens. Its contribution to fungal virulence varies among species, but it is considered an important determinant of virulence in *Candida* species [59]. It has been observed that *C. tropicalis* pmr1Δ/Δ and och1Δ/Δ mutants show increased exposure of both β1,3-glucan and chitin on the cell wall surface. The N-linked mannan core found on the surface of the *C. tropicalis* och1Δ null mutant was capable of inducing cytokine production, and a mannose-independent pathway for IL-10 production is present during the *C. tropicalis*–mononuclear cell interaction [60]. On the other hand, loss of Och1 in *C. parapsilosis* led to cell aggregation, morphogenesis failure, increased susceptibility to cell wall-perturbing agents, and defects in cell wall composition [61].

On the other hand, the deletion of genes encoding the Mnn2 family of α1,2-mannosyltransferases, which are involved in the synthesis of the outer chains of N-mannan, alters the addition of mannose side chains that are essential for the stabilization and extension of the central structure of N-mannan. It has been described that a reduction in N-mannan branching leads to decreased immune recognition, especially affecting the recognition by monocytes; furthermore, the complete elimination of the branched outer chains results in greater exposure of β-glucan and has been associated with attenuated virulence [62].

### 4.3. Cryptococcus neoformans

*Cryptococcus neoformans* is a highly adaptable fungal pathogen that employs multiple mechanisms to establish infections [63]. It is capable of escaping macrophages through vesicular encapsulation, a process known as non-lytic exocytosis or vomocytosis. In addition, the phagocytosed capsular component glucuronoxylomannan (GXM) can induce macrophage apoptosis via the Fas/FasL pathway and inhibit subsequent immune activation [64,65].

Furthermore, this fungus can evade immune surveillance by dendritic cells through the action of the virulence factor urease, which impairs dendritic cell maturation and antigen-presenting function. On the other hand, the cryptococcal capsule component GXM can exert chemotactic effects on neutrophils and promote the shedding of L-selectin and TNF-α from their cell surface. This process decreases neutrophil adhesion to endothelial cells and consequently limits their migration toward the site of infection [66].

Titan cells also play a role in immune evasion. These are *Cryptococcus* cells, induced by macrophages, that exceed 10 μm in diameter. It has been observed that the progeny derived from Titan cells are more resistant to hostile immune environments, such as oxidative and nitrosative stress, and display an enhanced ability to proliferate within phagocytes [67].

### 4.4. Histoplasma capsulatum

It has been demonstrated that this pathogen uses macrophages as a proliferative niche while largely avoiding the activation of host signaling receptors. *Histoplasma capsulatum* yeasts enter macrophages via binding to phagocytic receptors. Simultaneously, α-glucan masks the immunostimulatory β-glucans on the cell wall, while a secreted endoglucanase degrades any exposed β-glucans, thereby minimizing recognition by the host receptor Dectin-1 [68].

*Histoplasma capsulatum* yeasts have been shown to counteract reactive oxygen species produced by phagocytes through the expression of oxidative stress defense enzymes, including extracellular superoxide dismutase and extracellular catalase. Additionally, within the phagosome, *H. capsulatum* blocks phagosomal acidification, acquires essential metals such as iron and zinc, and employs de novo biosynthetic pathways to overcome nutritional limitations [69].

### 4.5. Blastomyces dermatitidis

*Blastomyces dermatitidis* is a pathogenic fungus that employs three key mechanisms to establish infection in the host. Overexpression of specific yeast-phase virulence factors, such as Blastomyces adhesin-1 (BAD1), has been shown to promote tissue adhesion, facilitate evasion of host immune defenses, and enable extrapulmonary dissemination. Additionally, during the mold-to-yeast transition, the β-(1,3)-D-glucan content of the cell wall decreases to less than 5%, thereby limiting recognition by host immune cells [8].

*Blastomyces yeast* cells also produce dipeptidyl peptidase IVA (DPPIVA), a serine protease that can reduce the activity of cytokines released by host immune cells. In particular, DPPIVA degrades granulocyte–macrophage colony-stimulating factor (GM-CSF), thereby impairing the recruitment of innate immune cells [70]. Moreover, *Blastomyces* yeasts are relatively resistant to reactive oxygen species produced by macrophages and neutrophils [71].

### 4.6. Coccidioides immitis

During endospore differentiation, *Coccidioides immitis* produces a metalloproteinase (Mep1) that digests an immunodominant cell surface antigen (SOWgp), which modulates the host immune response and leads to impaired cellular immunity to coccidioidal infection. This mechanism also prevents host recognition of endospores during their developmental phase [72,73]. Another important mechanism is the induction of elevated host arginase I production and fungal urease activity, both of which contribute to tissue damage at infection sites. Arginase I competes with inducible nitric oxide synthase (iNOS) in macrophages for their shared substrate, L-arginine, thereby reducing nitric oxide (NO) production. This, in turn, leads to increased synthesis of host ornithine, which promotes pathogen growth and proliferation by providing monoamines that are absorbed and utilized for polyamine synthesis via the parasite’s metabolic pathways [73].

### 4.7. Pneumocystis jirovecii

*Pneumocystis jirovecii* cells lack chitin and glucans during certain stages of their cell cycle, which may help avoid triggering innate and adaptive immune responses. In addition, surface antigenic variation plays a crucial role in evading the human immune system [74].

The major surface glycoprotein (gpA or MSG) is involved in both adhesion of *Pneumocystis* to host pulmonary epithelial cells and in immune evasion. Although multiple gpA/MSG isoforms are encoded in the genome of *P. carinii*, only one isoform is expressed at a given time, potentially allowing the organism to evade host defenses and establish infection [75].

### 4.8. Paracoccidioides brasiliensis

*Paracoccidioides brasiliensis* interferes with dendritic cell maturation by inhibiting prostaglandin E2 (PGE2) production, thereby preventing phenotypic changes and the proper secretion of cytokines essential for CD4^+^ T cell activation [76]. Additionally, intracellular *Paracoccidioides* species have demonstrated the ability to survive and even replicate within neutrophils, macrophages, giant cells, and dendritic cells. This intracellular lifestyle enables the fungus to disseminate to other tissues while shielded by phagocytic cells—a strategy known as the Trojan horse mechanism [77]. 

This fungus produces a glycoprotein known as gp43, which exerts a negative regulatory effect on macrophages by inhibiting the release of nitric oxide (NO) and hydrogen peroxide (H_2_O_2_) [78]. Additionally, extracellular vesicles from virulent *P. brasiliensis* strains have been shown to induce the expression of TLR4 and Dectin-1 in innate immune cells, while downregulating TLR2, CD80, CD86, and TNF-α. This results in reduced maturation of dendritic cells and macrophages, leading to a weaker immune response and facilitating fungal immune evasion [79].

### 4.9. Mucorales

*Mucorales* employ several mechanisms to evade the immune system, including blocking phagosomal maturation and utilizing species-specific survival strategies within macrophages. For instance, *Rhizopus* species can persist as dormant spores inside macrophages, while *Mucor* species tend to germinate and escape [80]. It has also been shown that *Mucorales* can suppress nitric oxide (NO) accumulation in macrophages stimulated with lipopolysaccharide and interferon-gamma, despite robust expression of *Nos2* mRNA and inducible nitric oxide synthase (iNOS) protein [81].

### 4.10. Fonsecaea pedrosoi

Da Silva et al. observed that the conidia of this fungus inhibit the maturation of Langerhans cells [82]. Experimental studies have demonstrated that the transformation of this fungus into sclerotic cells impairs Th1 cytokine production during the early stages of infection and alters neutrophil-derived reactive oxygen species generation [83]. It has also been shown that melanized *Fonsecaea pedrosoi* cells are more resistant to both H_2_O_2_ and nitric oxide (NO) [84].

### 4.11. Sporothrix schenckii

Asteroid bodies represent one of the immune evasion strategies employed by *Sporothrix schenckii*. These structures consist of yeast-like cells coated with IgG and IgM molecules, which may help camouflage the fungal cells from immune recognition by host cells [85]. In addition, *S. schenckii* secretes proteases capable of degrading various IgG subclasses, further contributing to immune evasion. The lipid components of *S. schenckii* have been shown to induce and sustain inflammation through Toll-like receptor 4 (TLR4) or inflammasome activation, while fungal exoantigen recognition via TLR2 may also act as an immune evasion mechanism, potentially prolonging and exacerbating infection [86]. Moreover, soluble fungal surface antigens (SolAgs) and lipid antigens (LipAgs) have been observed to impair phagocytosis and the production of immune mediators [87].

The cell wall peptide known as rhamnomannan is a key component used by fungi to suppress the immune response, particularly when immune cells are exposed to it for prolonged periods. This prolonged exposure may lead to immune tolerance against *Sporothrix schenckii* [85]. Lozoya-Pérez et al. silenced *S. schenckii* OCH1, after which they observed changes in both the composition of the cell wall and the exposure of inner components on the surface. The mutant showed a reduced capacity to stimulate TNF-α and IL-6, but induced higher levels of IL-10 [59].

### 4.12. Talaromyces marneffei

The Mp1p protein is a key virulence factor that may help *Talaromyces marneffei* evade elimination by the immune system [88]. The NCOR2-013 protein has been identified as forming a corepressor complex with TBL1XR1/TBLR1 and HDAC3, thereby inhibiting JunB-mediated transcriptional activation of proinflammatory cytokines by suppressing histone acetylation. Moreover, it evades macrophage-mediated destruction through TUT1-mediated alternative splicing of NCOR2/SMRT [89].

When conidia are phagocytosed by alveolar macrophages and polymorphonuclear neutrophils, they rapidly undergo a morphogenetic transition into the yeast form, multiply intracellularly by binary fission, and express the yeast-phase-specific antigen within 24 h post-phagocytosis. Instead of being eliminated through the macrophage’s natural clearance mechanisms, they persist by avoiding lysosomal fusion and escape into the cytoplasmic environment [90]. It has been demonstrated that *Talaromyces marneffei* depletes levels of arachidonic acid (AA), a key proinflammatory lipid mediator, to evade the host’s innate immune defense. This occurs via the secreted mannoprotein Mp1p, which binds to AA, sequestering this critical proinflammatory signaling lipid. As a result, AA, its downstream metabolites, and the cytokines interleukin-6 and tumor necrosis factor-α are downregulated in macrophages [91].

### 4.13. Malassezia spp.

The lipid-rich microfibrillar layer surrounding *Malassezia* may help shield the yeast cells from triggering inflammatory responses in the host. Kesavan et al. demonstrated that lipid extraction restored the ability of yeast cells to induce proinflammatory cytokine production [92]. In the case of *Malassezia furfur*, it has been revealed that the fungus is capable of converting tryptophan into a variety of indole alkaloids. Indoles derived from *M. furfur*, such as malassezin, indirubin, and indolo [3,2-b]carbazole (ICZ), act as potent ligands for the host’s aryl hydrocarbon receptor (AhR), thereby potentially altering the function of all epidermal cells expressing this receptor. Some tryptophan metabolites may promote melanocyte apoptosis or inhibit the respiratory burst in neutrophils [93]. *Malassezia* can also modulate immune responses by interacting with melanocytes, fibroblasts, keratinocytes, and dendritic cells [94].

### 4.14. Trichophyton spp.

García-Madrid et al. analyzed the expression of Toll-like receptors TLR2, TLR4, and TLR6, as well as the production of human β-defensins (HBD)-1 and HBD-2, interleukin IL-1β, and IL-8 in primary keratinocyte cultures exposed to *Trichophyton rubrum*. The results revealed changes in the size and granularity of keratinocytes stimulated with whole conidia, along with a decrease in the expression of TLR2 and TLR6. A reduction in the production of HBD-1 and HBD-2 was also observed. These findings indicate that keratinocytes are seemingly manipulated by the fungus to promote its establishment, persistence, and survival [95]. *T. rubrum* is also able to evade mannose receptors on dendritic cells and macrophages [96].

### 4.15. Microsporum spp.

The production of aspartyl proteases has been associated with the degradation of host immune system proteins. Additionally, the presence of catalases has been observed, which protect the fungi from reactive oxygen species produced as part of the host’s immune defenses [97].

*Microsporum canis* forms a biofilm composed of a network of organized and interconnected hyphae embedded in an extracellular polysaccharide matrix. The biofilm acts as a protective barrier that limits the penetration of antifungal agents, facilitates the emergence of tolerant fungal cells, and decreases the interaction between the fungus and the host immune system [97].

Table 1 presents a comprehensive summary of the virulence mechanisms employed by medically significant fungal species; by synthesizing these key pathogenic traits, the table offers valuable insight into how various fungal species colonize, invade, and persist within human hosts, contributing to disease progression and immune system evasion.

## 5. Commensal Relationship Between Host and Fungi 

Fungal symbionts vary in their route of acquisition and the niches they inhabit. For example, colonization by *Candida albicans* occurs within the first months of life, with the primary sites of colonization being the mucosal surfaces of the oral cavity and the intestine. In the intestine, the thick mucus layer effectively separates *C. albicans* from the epithelial cells. Moreover, *C. albicans* exists as a commensal on mucosal surfaces predominantly in yeast form, which is not easily recognized by epithelial cells [108,109]. In contrast, in the oral cavity, commensalism is maintained by nonspecific barriers such as saliva and antimicrobial peptides [108]. Additionally, the presence of commensal bacteria, secretory IgA, and surveillance by IL-17-producing T cells contribute to a low fungal burden and, consequently, the absence of invasive tissue damage [109]. On the other hand, *Saccharomyces cerevisiae* has been described as having the ability to stimulate the immune system to mount more effective responses against pathogens, as it can induce trained immunity in monocytes through increased production of TNF-α and IL-6 [110].

## 6. Antifungal Drug Resistance: Mechanisms, Challenges, and Threats

### 6.1. Molecular Basis and Clinical Emergence of Azole Resistance in Fungal Pathogens

These antifungal agents disrupt ergosterol synthesis by inhibiting the cytochrome P450-dependent enzyme lanosterol 14-α-demethylase, which is encoded by *ERG11* in yeasts and by *CYP51A/CYP51B* in molds. Azole resistance can arise through both acquired mechanisms and intrinsic characteristics of the fungal species [111,112]. Currently, there is a steady emergence of fluconazole-resistant isolates of *C. glabrata*, *C. auris*, and *C. tropicalis* [113].

Among the main mechanisms of resistance to these drugs is the overexpression or alteration of the *ERG11/CYP51A/CYP51B* genes. When *ERG11* is overexpressed, an increase in ergosterol biosynthesis has been observed, resulting from the elevated production of lanosterol 14α-demethylase. On the other hand, amino acid substitutions in *ERG11* can lead to an enzyme with reduced binding affinity to azoles [111,112]. 

Overexpression of ERG11 has been associated with gain-of-function mutations in the transcriptional activator UPC2; this has been observed in azole-resistant *C. albicans* [114]. Mutations in the *CYP51A* gene are one of the main mechanisms of azole resistance in *Aspergillus* [112]. Loss-of-function mutations affecting other enzymes involved in ergosterol biosynthesis, such as the Δ-5,6-desaturase Erg3, may also promote the development of resistance to azole antifungal agents [111]. ATP-binding cassette (ABC) transporters, including *Cdr1* and *Cdr2*, along with the major facilitator transporter *Mdr1*, have been associated with clinical azole resistance across several *Candida* species. In *C. albicans*, mutations in the transcription factor gene *MRR1* can increase *MDR1* expression, contributing to the resistant phenotype observed in azole-resistant isolates [111,115].

### 6.2. Echinocandin Resistance in Candida: Target Site Mutations and Adaptive Compensation

These drugs cause a decrease in the synthesis of 1,3-β-D-glucan, a component of the fungal cell wall, because they act on the enzyme 1,3-β-D-glucan synthase [112]. Resistance to these drugs occurs through amino acid substitutions in regions of the Fks subunits of glucan synthase, as seen in mutations in the *FKS1* gene. It has been reported that some *Candida* species possess natural polymorphisms in the *FKS* genes, resulting in inherently higher minimum inhibitory concentrations (MICs) against echinocandins. For example, *C. parapsilosis* carries the P660A substitution, while *C. guilliermondii* exhibits the L633M and T634A substitutions, all of which contribute to reduced susceptibility to echinocandins [111,112].

The signaling pathways that maintain cell wall integrity mediated by protein kinase C (PKC), the phosphatase calcineurin, and the molecular chaperone Hsp90 are crucial for fungi to tolerate echinocandin treatments. When these pathways are activated, the fungus triggers compensatory responses, such as increasing chitin production. This happens because echinocandins inhibit the enzyme 1,3-β-D-glucan synthase, reducing glucan synthesis. To counterbalance this, the fungus boosts chitin synthesis, which helps it become less susceptible to these drugs [111,112].

Polyenes bind to ergosterol, altering the permeability of the cell membrane and causing leakage of intracellular contents. Resistance to amphotericin B can result from a decrease in the amount of ergosterol present in the fungal cell membrane, reducing the drug’s ability to exert its antifungal effect. Organisms with intrinsically reduced susceptibility to amphotericin B include species of *Aspergillus* such as *A. terreus*, *A. flavus*, and *A. nidulan* [112].

## 7. Immunomodulatory and Novel Therapeutic Approaches in Antifungal Treatment

Currently, various immunomodulatory agents are being studied with the aim of being used as complementary therapies to antifungals, since this dual strategy can stimulate the immune response, especially in immunocompromised individuals, thereby achieving the eradication of fungal infections [25].

Cytokine-based therapy aims to stimulate the proliferation, differentiation, and activation of immune cells. Among these therapies, colony-stimulating factors (CSFs) are particularly notable. These include macrophage colony-stimulating factor (M-CSF), which specifically promotes the proliferation and activity of macrophages; granulocyte colony-stimulating factor (G-CSF), which stimulates the proliferation, survival, and function of neutrophils; and granulocyte–macrophage colony-stimulating factor (GM-CSF), which acts on a broad range of myeloid cells, including dendritic cells (DCs), macrophages, neutrophils, monocytes, megakaryocytes, and eosinophils [25].

The use of interleukins is also being widely studied. IL-17 plays an important role in protection against *C. albicans*. Recombinant IL-12 has shown synergistic activity with fluconazole in neutropenic mice, thereby providing protection against invasive candidiasis. Additionally, IL-23 has been identified as a key element in the immune response to chronic fungal infections [116,117,118].

The administration of short courses of IFN-γ, together with amphotericin B, improves the clearance of *C. neoformans* from the cerebrospinal fluid in patients with HIV. Additionally, IFN-γ is effective in controlling disseminated infections caused by *Candida* and *Aspergillus*. On the other hand, treatment with TNF-α exerts a protective effect against systemic and pulmonary infection by *A. fumigatus* in both wild-type and neutropenic mice [25]. Another promising strategy involves the use of immune checkpoint inhibitors, which block inhibitory signals that dampen immune responses, thereby enhancing the activity of immune cells against fungal pathogens [119].

The use of T cells represents a strategy that involves transferring these cells from a healthy donor to an immunocompromised patient in order to strengthen the immune defense. T cells can stimulate the production of IFN-γ and TNF-α, as they act as modulators and effectors of the Th1 response, which is initiated through TLR4 signaling; this helps eliminate pathogenic fungi. In contrast, Th2 responses mediated by TLR2 lead to the production of anti-inflammatory cytokines such as IL-4 and IL-10, which regulate inflammation but compromise antifungal resistance [119]. However, this technique faces several challenges. These include the low frequency of fungus-specific T cells, which complicates large-scale expansion, the potential development of graft-versus-host disease (GVHD), and the use of immunosuppressive agents as GVHD prophylaxis, which may impair the function of the infused T cells [25].

Vaccines are considered one of the most cost-effective and successful methods for eradicating infectious diseases. The subunit vaccine known as PEV7 has demonstrated, in animal model studies (rats), the ability to stimulate the production of specific IgG and IgA antibodies to provide protection against vaginal candidiasis [25]. Pevion, a Swiss biotech company, conducted a Phase I clinical trial of PEV7 to evaluate its safety and immunogenicity in 48 healthy female volunteers. The results showed a favorable safety profile and the induction of specific and functional B cell memory in 100% of vaccinated women, which was highly promising in terms of the vaccine’s therapeutic potential [120]. 

The NDV-3 vaccine, composed of the *C. albicans* agglutinin-like sequence 3 protein (Als3p), conferred protection in mice against *C. albicans* infection [25]. A Phase I clinical trial in humans enrolled 40 healthy adults who were randomized to receive a single dose of NDV-3 containing either 30 or 300 μg of Als3p, or a placebo. The results showed that total anti-Als3p IgG and IgA1 levels for both doses peaked on day 14 post-vaccination, with 100% seroconversion among all vaccinated subjects. Additionally, six months after the initial dose, 19 participants received a second dose identical to their first to assess memory B and T cell immune responses. The second dose resulted in a significant increase in IgG and IgA1 titers in over 70% of subjects, with the greatest response observed in those who had received the 30 μg dose [121].

In a randomized, double-blind, placebo-controlled Phase 2 trial involving 188 women, the results revealed that a single intramuscular dose of NDV-3A was safe and induced rapid and robust immune responses from both B and T cells. Additionally, a statistically significant increase in the percentage of patients without symptoms was observed 12 months post-vaccination (42% vaccinated vs. 22% placebo; P = 0.03), along with a doubling of the median time to the first symptomatic episode (210 days in vaccinated vs. 105 days in placebo) [122]. Regarding *Cryptococcus*, Stempinski et al. observed that three doses of the EPS-CRM 197 vaccine produced greater survival compared with unvaccinated mice [123]. Meanwhile, Normile and Del Poeta reported that the HK Δsgl1 vaccine demonstrated significant efficacy and clinical relevance by providing robust protection to the host against cryptococcosis in three vaccination models, even under conditions of CD4 cell deficiency [124].

The recombinant peptide vaccine NXT-2, based on a conserved KEX1 sequence found in multiple pathogenic fungi, has been shown to be effective in protecting murine and non-human primate models against invasive aspergillosis, systemic candidiasis, and pneumocystosis. Additionally, a β-glucan–CRM197-based vaccine was able to elicit protective immune responses in mice challenged with *C. albicans* and *A. fumigatus* [25].

## 8. Conclusions

Fungal infections constitute a growing global health challenge, particularly affecting immunocompromised individuals and populations in low- and middle-income countries. Pathogenic fungi have developed complex immune evasion strategies such as cell wall remodeling, biofilm formation, colonization of immune-privileged niches, and modulation of both innate and adaptive immunity that enable them to persist, disseminate, and evade host defenses.

The situation is further exacerbated by the rapid emergence of antifungal resistance across all major drug classes, significantly limiting therapeutic options. Multidrug-resistant species like *Candida auris* highlight the urgent need for enhanced surveillance, new antifungal agents, and immunomodulatory approaches. Precision immunotherapies and vaccine platforms targeting conserved fungal antigens offer promising advances in overcoming current treatment limitations.

Incorporating invasive fungal infections into national and global infectious disease control agendas is essential, especially in resource-limited settings where environmental exposure, healthcare access barriers, and syndemic conditions amplify the burden. Within a One Health framework, the intersections of human, animal, and environmental health intensified by climate change, urbanization, and deforestation demand a holistic approach to prevention and preparedness.

Advancing our understanding of fungal immune evasion and host–pathogen interactions is critical not only for improving diagnostics and clinical outcomes but also for guiding global strategies in fungal disease prevention, resilience, and equitable health policy implementation.

## Figures and Tables

**Figure 1:**
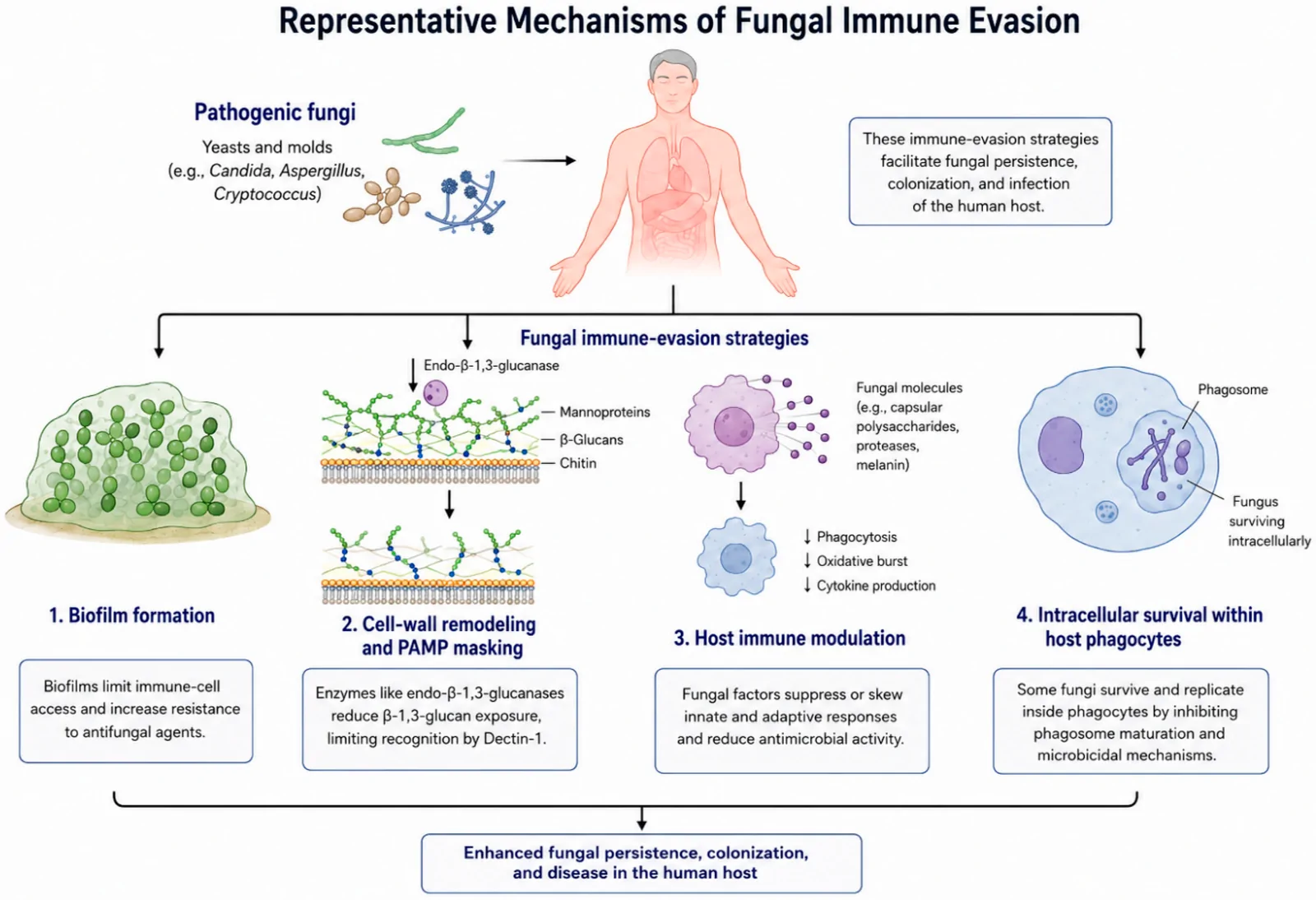
Main mechanisms of immune system evasion in fungi.

**Figure 2:**
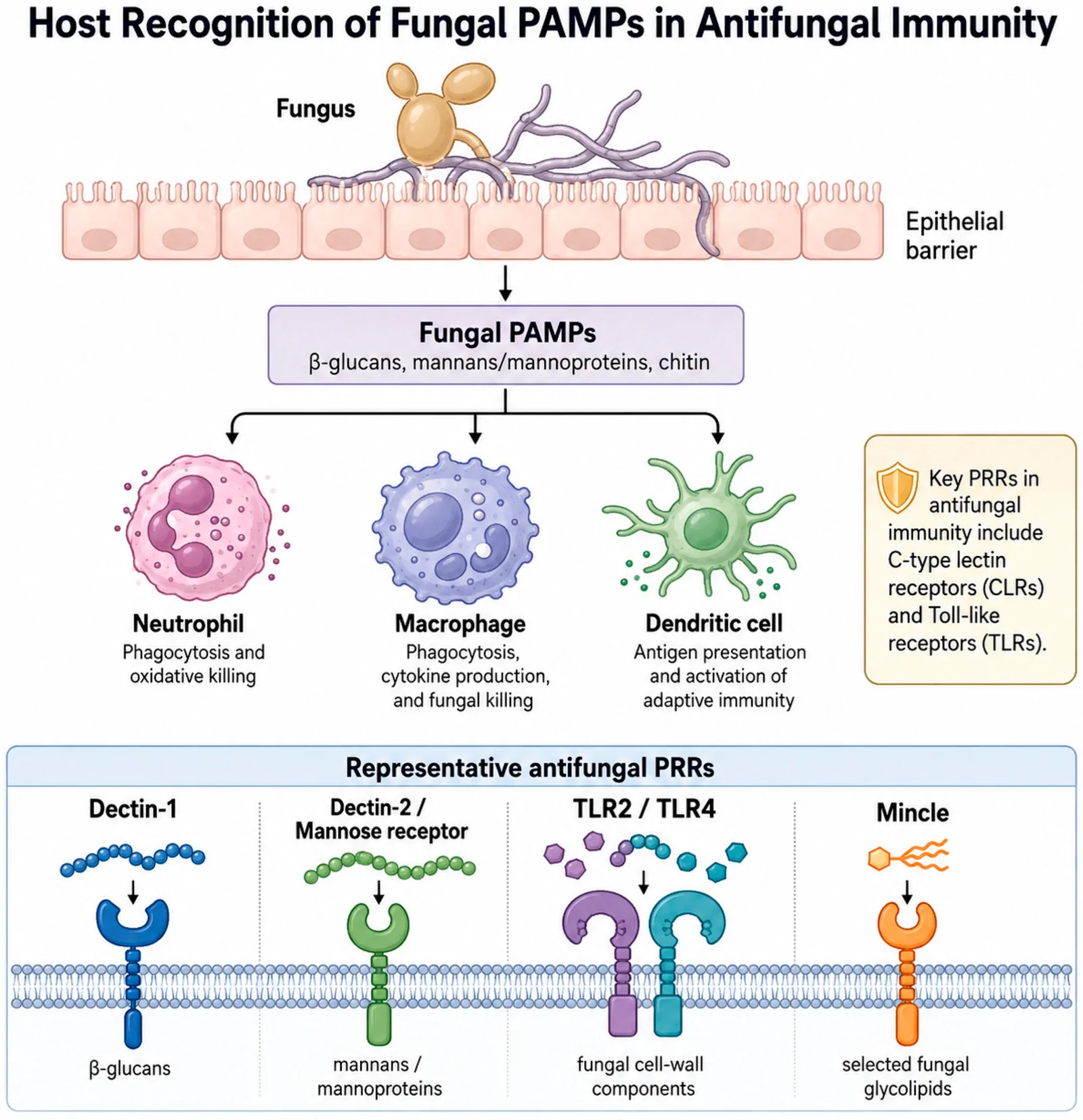
Innate immunity against mycoses.

**Figure 3:**
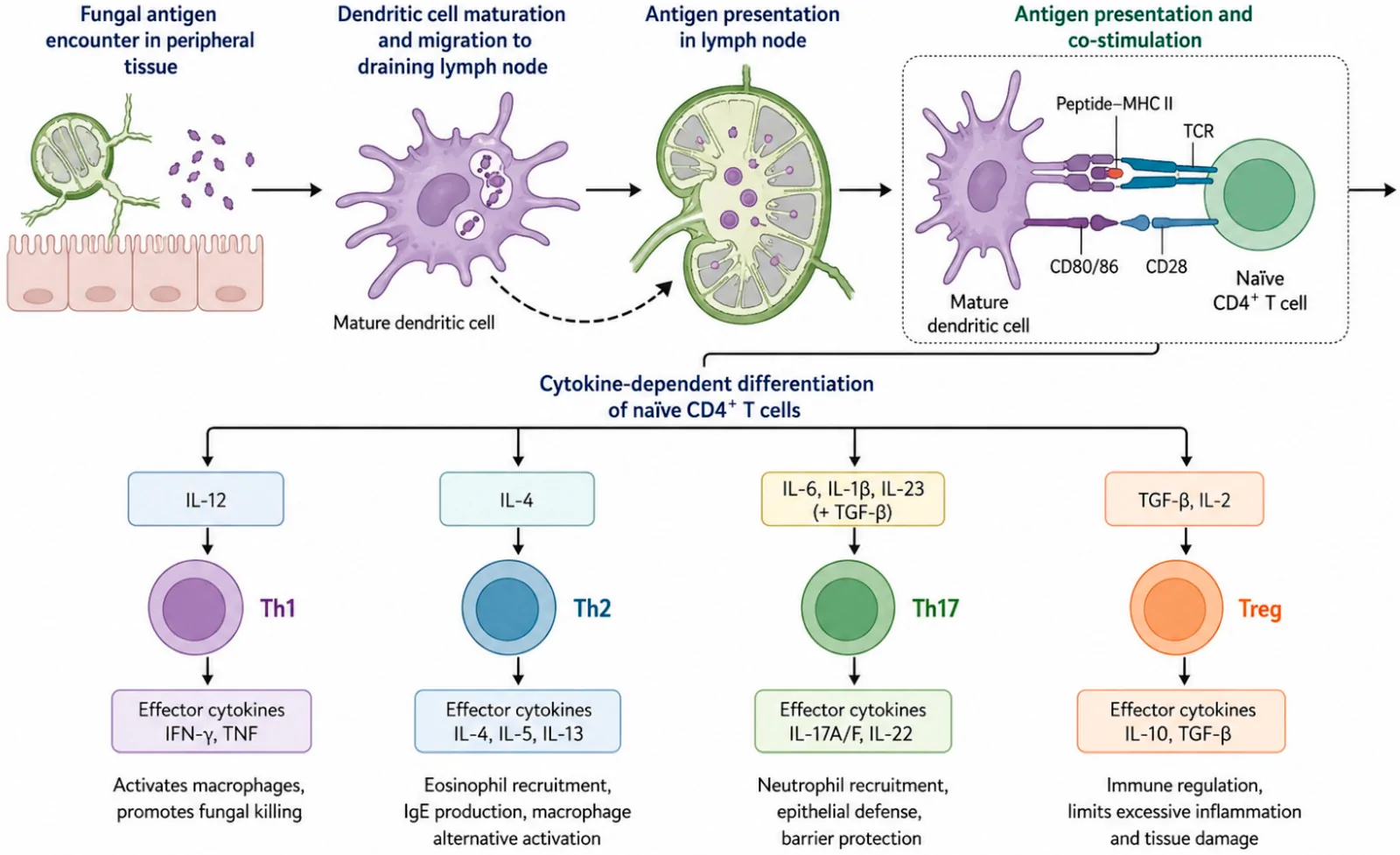
Adaptative immunity against fungal infections.

**Figure 4:**
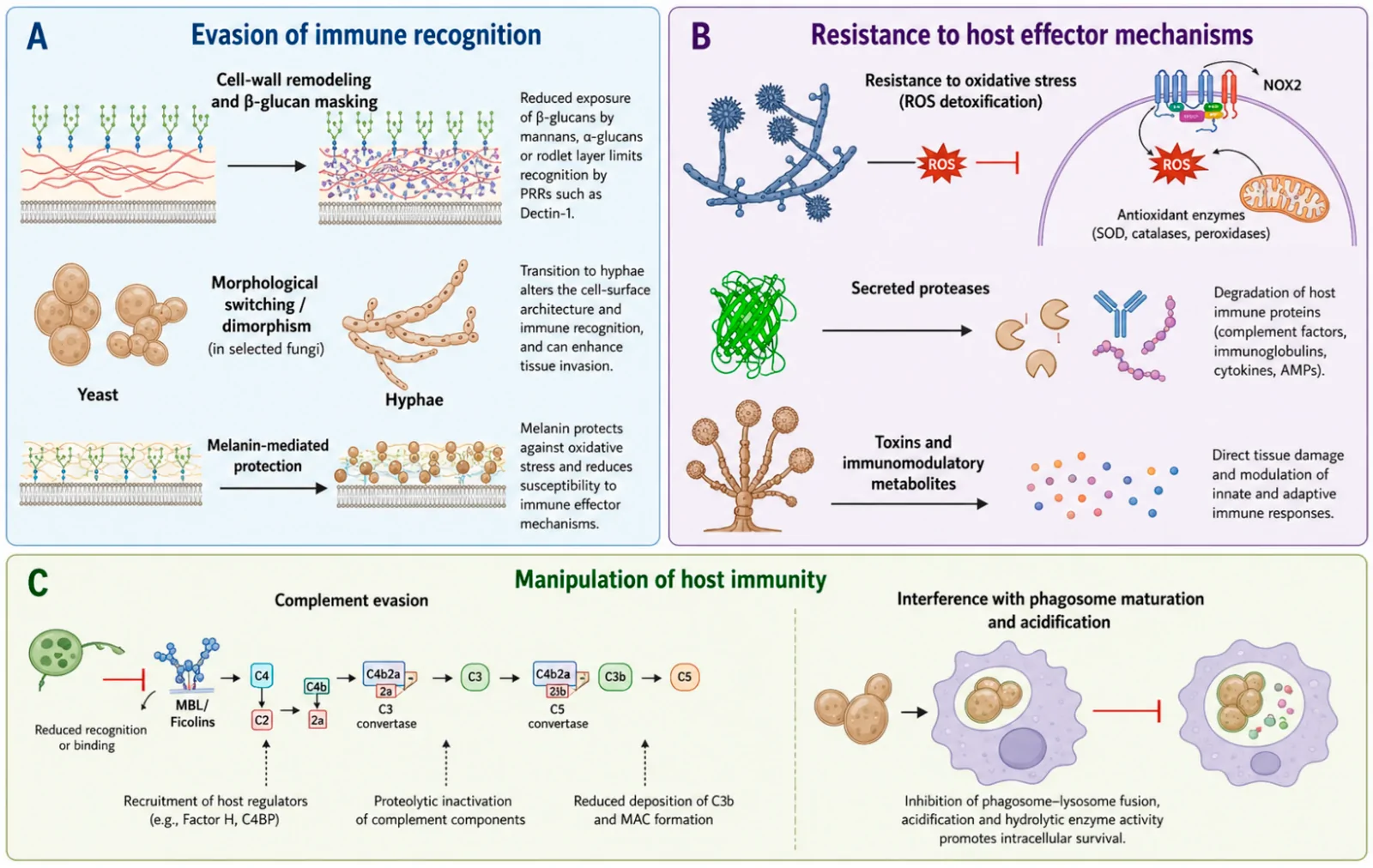
Mechanisms of phagocytic action inhibition that promote fungal intracellular survival. (**A**) Stealth: Fungi reduce immune detection by masking β-glucans, transitioning from yeast to hyphae, and producing melanin. (**B**) Attack: Pathogenic fungi counterattack the immune mechanisms by neutralizing ROS, degrading host proteins, and producing toxins. (**C**) Control: Fungi are capable of manipulating immune responses by inhibiting phagosome maturation, modulating cytokines, and preventing complement activation.

**Table 1:** Synthesized overview of virulence mechanisms in medically important fungi.

Fungus	Virulence Factors	References
*Aspergillus* spp.	β-(1,3)-glucan, galactomannan (Afm1p and Afm2p), chitin synthase, α-(1,3)-glucan synthase, melanin, and mycotoxins	[98]
*Candida*	The morphological transition from blastospores to hyphae, adhesins (Als3 and Hwp1), biofilm formation, hydrolytic enzymes, and thigmotropism	[99]
*Cryptococcus neoformans*	Adaptation to the host environment, phenotypic variations, and degradative enzymes such as proteases and lipases.	[100]
*Histoplasma capsulatum*	Surviving at host temperature, ability to persist within macrophages, evasion of reactive oxygen and nitrogen species, regulation of phagosome acidification, and prevention of lysosomal fusion	[101]
*Blastomyces dermatitidis*	Adhesin-1, dipeptidyl peptidase IVA (DPPIVA), genes involved in zinc uptake	[70]
*Coccidioides*	Production of a dominant glycoprotein from the outer wall of the spherule (SOWgp), which modulates the host immune response, along with increased production of host-derived arginase I and *Coccidioides* urease, both of which contribute to tissue damage.	[73]
*Pneumocystis jirovecii*	Surface antigenic variation, surface glycoprotein	[74,75]
*Paracoccidioides brasiliensis*	Cell adhesion molecules and the presence of melanin pigments.	[102]
*Mucorales*	Two distinct strategies for iron acquisition: a high-affinity iron uptake system and the production of siderophores, dimorphism.	[103]
*Fonsecaea pedrosoi*	Ability to alternate between saprophytic and pathogenic forms, formation of extracellular vesicles capable of modulating the production of proinflammatory and anti-inflammatory cytokines, as well as nitric oxide by macrophages	[104]
*Sporothrix schenckii*	Morphological transition, melanin, adhesins, extracellular vesicles, and glycoproteins in its cell wall.	[105]
*Talaromyces marneffei*	The dimorphic switch; enzymes that detoxify reactive oxygen species; heat shock proteins (HSPs); and the Mp1p protein.	[88,90]
*Malassezia* spp.	Cell wall resistance, lipases, phospholipases, acid sphingomyelinases, melanin, reactive oxygen species (ROS), indoles, hyphae formation, hydrophobicity, and biofilm formation.	[106]
*Trichophyton* spp.	Several enzymes, including keratinase, protease, lipase, deoxyribonuclease, elastase, and collagenas	[107]
*Microsporum* spp.	Main virulence factors are keratinases, fungalysins, and subtilisins.	[97]

## Data Availability

No new data were created or analyzed in this study. Data sharing is not applicable to this article.

## Abbreviations

The following abbreviations are used in this manuscript:

PAMPs	Pathogen-associated molecular patterns
PRRs	Pattern recognition receptors
TLRs	Toll-like receptors
TIR	Toll/IL-1 receptor
MR	Mannose receptor
ROS	Reactive oxygen species
NADPH	Nicotinamide adenine dinucleotide phosphate
TH	T helper
NETs	Neutrophil extracellular traps
SOD	Superoxide dismutase
CAT	Catalase
PRX	Peroxiredoxin
PMN-MDSC	Polymorphonuclear myeloid-derived suppressor cell
MDSCs	Myeloid-derived suppressor cells
PBMCs	Peripheral blood mononuclear cells
GXM	Glucuronoxylomannan
FH	Factor H
FHL-1	Factor H-like protein 1
FHR-1	Factor H-related protein 1
C4BP	C4b-binding protein
